# Non-genomic mechanisms mediate androgen-induced PSD95 expression

**DOI:** 10.18632/aging.101913

**Published:** 2019-04-20

**Authors:** Yizhou Zhang, Sha Li, Huan Chen, Meiqin Chen, Shixiong Mi, Jingle Ma, Chang Wang, Hongmei Sun, Xiaoyun Liu, Huixian Cui

**Affiliations:** 1 Department of Anatomy, Hebei Medical University, Shijiazhuang, China; 2 Neuroscience Research Center, Hebei Medical University, Shijiazhuang, China; 3 Grade 2016, Clinical Medicine Specialty, Hebei Medical University, Shijiazhuang, China; 4 Neurology Department, The Second Hospital of Hebei Medical University, Shijiazhuang, China; * Equal contribution

**Keywords:** androgen, non-genomic action, ZIP9, synaptic plasticity, PSD95

## Abstract

The non-genomic actions of androgen-induced synaptic plasticity have been extensively studied. However, the underlying mechanisms remain controversial. We recently found that testosterone-fetal bovine serum albumin (T-BSA), a cell membrane-impermeable complex, led to a rapid increase in the postsynaptic density 95 (PSD95) protein level through a transcription-independent mechanism in mouse hippocampal HT22 cells. Using T-BSA conjugated FITC, we verified the presence of membrane androgen-binding sites. Here, we show that T-BSA-induced PSD95 expression is mediated by G-protein-coupled receptor (GPCR)-zinc transporter ZIP9 (SLC39A9), one of the androgen membrane binding sites, rather than the membrane-localized androgen receptor. Furthermore, we found that T-BSA induced an interaction between ZIP9 and Gnα11 that lead to the phosphorylation of Erk1/2 MAPK and eIF4E, which are critical in the mRNA translation process. The PSD95 and p-eIF4E expression decreased when knockdown of ZIP9 or Gnα11 expression or inhibition of Erk1/2 activation. Taken together, these findings suggest that ZIP9 mediates the non-genomic action of androgen on synaptic protein PSD95 synthesis through the Gnα11/Erk1/2/eIF4E pathway in HT22 cells. This novel mechanism provides a theoretical basis to understand the neuroprotective mechanism of androgen.

## INTRODUCTION

Age-related hormonal changes are associated with cognitive impairment in various neurodegenerative diseases such as Alzheimer’s disease (AD) [1, 2]. It has been shown that androgen deficiency contributes to the development of AD in elderly men [3, 4]. Serologic tests have shown that men with AD have reduced levels of total or bioavailable testosterone, a major endogenous androgen, while testosterone supplementation could improve their cognitive functions to various degrees, with a particularly notable improvement in spatial memory [5–8].

Animal and cellular models have been used to explore the mechanism underlying this relationship between androgen deficiency and AD. In this regard, androgen deficiency leads to an increase in β-amyloid plaques and neurofibrillary tangles, which are the 2 major pathological hallmarks in AD patients [1]. Moreover, androgen deficiency-caused alteration of hippocampal synapses is another important factor of AD. Previous studies have also demonstrated that testosterone supplementation rapidly increases the dendritic spine density of hippocampal neurons in male rats [9–11]. Our previous *in vivo* study has revealed that testosterone rapidly increases PSD95 expression along with dendritic spine density in the hippocampus of the senescence-accelerated mouse prone 8 (SAMP8) line [12], which is a naturally occurring mouse line that displays a phenotype of accelerated aging. The synaptic protein PSD95 is required for synaptic maturation [13, 14] and dendritic spine formation and stabilization [15]. Meanwhile, increasing the expression of Dlg4/PSD95 through epigenetic mechanisms rescued learning and memory deficits in aged and Alzheimer’s disease mice [16].

According to the classical androgen action, testosterone enters cells through the plasma membrane and binds to androgen receptors (ARs). The androgen-bound receptors in the cytoplasm or nucleus translocate to the nucleus and act on specific DNA responsive elements to regulate the transcription of target genes and usually results in alteration of mRNA and protein synthesis to ultimately affect cellular biology [17, 18]. However, this genomic mechanism is unlikely to be fully responsible for some rapid biological responses to androgen, implying that androgen produces a potential non-genomic action [19]. Indeed, the non-genomic actions of androgen occur in a time frame of seconds to minutes, suggesting that they are independent of the AR-mediated transcription/translation mechanisms [17]. In addition, membrane-impermeable steroid conjugates such as testosterone-fetal bovine serum albumin (T-BSA) can also perform these rapid actions, which provides strong evidence for a mechanism of membrane binding sites-initiated signaling. The reported androgen membrane-binding sites mainly include membrane-localized AR [20] and the novel G protein-coupled receptor (GPCR)-zinc transporter ZIP9 (SLC39A9), which directly interacts with the G-protein Gnα11 [21]. However, their roles in altering PSD95 expression in response to androgen are not clear.

In this study, we examined the effect of membrane-impermeable steroid T-BSA on PSD95 expression via transcription-independent mechanisms in mouse hippocampal HT22 cells. HT22 or its parents cell line HT4 are hippocampal neuronal cell lines that have been used as good model for memory-related studies because they are capable of mimicking long-term potentiation without establishing synaptic connections [22–25]. By using morphological analysis and molecular biology approaches, we determined a critical role for the novel membrane androgen binding site ZIP9 in mediating non-genomic action of androgen on PSD95 expression, rather than the membrane-localized AR. Furthermore, we identified the signaling pathway that mediates the androgen effect on PSD95 expression.

## RESULTS

### T-BSA rapidly increased PSD95 expression via membrane binding sites for androgen

**Figure 1:**
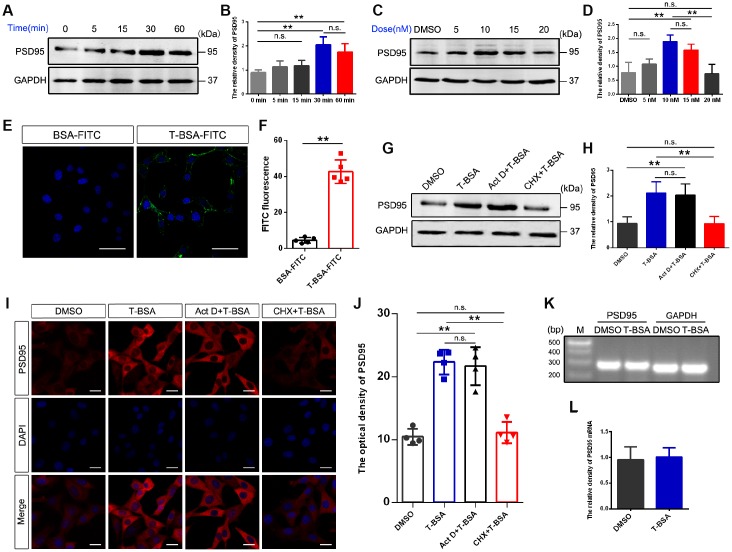
**T-BSA rapidly increased PSD95 expression through a transcription-independent mechanism**. (**A** and **B**) Time-dependent effects of T-BSA (0 min, 5 min, 15 min, 30 min and 60min) on PSD95 protein levels (n=5). (**C** and **D**) Dose-dependent effects of T-BSA (DMSO, 5 nM, 10 nM, 15 nM and 20 nM) on PSD95 protein levels (n=5). (**E** and **F**) FITC signals on the HT22 cell plasma membrane (n=5, scale bars = 50 μm). (**G** and **H**) Western blotting for PSD95 expression induced by T-BSA in HT22 cells pre-treated with 10 μM Act D or 200 μM CHX for 2 h (n=5). (**I** and **J**) Immunofluorescence staining for PSD95 induced by T-BSA in HT22 cells pre-treated with Act D or CHX (n=4, scale bars = 20 μm). (**K** and **L**) RT-PCR for PSD95 mRNA level induced by T-BSA (n=5). (n.s.: non-significant; * *P* < 0.05; ** *P* < 0.01).

To assess the impact of membrane-impermeable T-BSA on PSD95 expression, we first analyzed its time- and dose-dependent effects in HT22 cells. Western blotting analysis showed that treatment with 10 nM T-BSA (soluble in DMSO) significantly increased PSD95 protein expression at 30 min and 60 min but did not change the expression levels at 0 min, 5 min and 15 min (Figure 1A and 1B). T-BSA treatment significantly increased PSD95 expression at concentrations of 10 nM and 15 nM compared with DMSO (the vehicle group), 5 nM T-BSA and 20 nM T-BSA groups (Figure 1C and 1D). Given that incubation with 10 nM T-BSA for 30 min significantly increased PSD95 expression, this treatment condition was used in the following experiments.

We further verified the presence of membrane androgen-binding sites by using T-BSA conjugated FITC. The non-specific binding of T-BSA-FITC was evaluated using BSA conjugated FITC lacking a testosterone moiety. The binding of BSA-FITC was found to be negligible, so that non-specific binding via the BSA or FITC moiety of the T-BSA-FITC molecule can be excluded (Figure 1E and 1F).

### T-BSA increased PSD95 expression in a transcription-independent manner

To determine whether the effect of T-BSA on PSD95 expression depends on a transcription and/or translation process, we measured PSD95 protein level in HT22 cells after pre-treatment with the transcription inhibitor Act D or the translation inhibitor CHX. Western blotting and immunofluorescent staining analysis showed that CHX effectively inhibited the effect of T-BSA on PSD95 expression compared with T-BSA group. However, PSD95 expression had no significant change after treatment with Act D (Figure 1G–1J). Moreover, RT-PCR analysis revealed that T-BSA treatment did not affect the PSD95 mRNA level compared with DMSO treatment (Figure 1K and 1L).

### T-BSA affected PSD95 expression via ZIP9 but not membrane-localized AR

**Figure 2:**
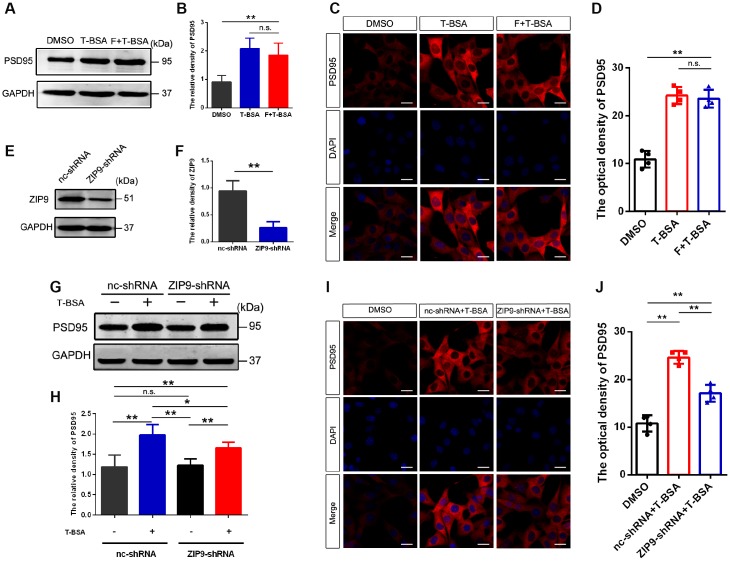
**T-BSA increased PSD95 expression via ZIP9 rather than membrane-localized AR.** (**A** and **B**) Western blotting for PSD95 expression induced by T-BSA in HT22 cells pre-treated with flutamide (F, 100 μM) for 1 h (n=5). (**C** and **D**) Immunofluorescence staining for PSD95 induced by T-BSA in HT22 cells pretreated with flutamide (n=4, scale bar = 20 μm). (**E** and **F**) Western blotting for the knockdown efficiency of ZIP9 protein in HT22 cells transfected with nc-shRNA or ZIP9-shRNA (n=5). (**G** and **H**) Western blotting for PSD95 expression induced by T-BSA in HT22 cells pre-treated with nc-shRNA or ZIP9-shRNA (n=5). (**I** and **J**) Immunofluorescence staining for PSD95 induced by T-BSA in HT22 cells pre-treated with nc-shRNA or ZIP9-shRNA (n=4, scale bars = 20 μm). (n.s.: not-significant; * *P* < 0.05; ** *P* < 0.01).

Since the results described above suggested that T-BSA increased PSD95 expression through non-genomic action, we next sought to identify whether these effects are mediated by membrane-localized AR and/or ZIP9. The AR antagonist flutamide was used to competitively bind AR to prevent them from functioning normally. Western blotting and immunofluorescent staining analysis revealed that PSD95 protein level in T-BSA treatment did not significantly differ from that in HT22 cells treated with flutamide plus T-BSA treatment (Figure 2A–2D).

To determine the role of ZIP9 in the upregulation of PSD95 induced by T-BSA, we then transfected HT22 cells with ZIP9-shRNA or negative control shRNA (nc-shRNA). The protein expression of ZIP9 in ZIP9-shRNA group was markedly lower than that in nc-shRNA group (Figure 2E and 2F). Western blotting and immunofluorescent staining analysis showed that ZIP9-shRNA treatment significantly reduced T-BSA-induced PSD95 levels compared with nc-shRNA plus T-BSA group (Figure 2G–2J). These data suggest that ZIP9 mediates the non-genomic action of T-BSA on PSD95 expression in HT22 cells, while the membrane-localized AR is not involved in this process.

### Formation of ZIP9/Gnα11 complex is required in T-BSA-induced PSD95 expression

**Figure 3:**
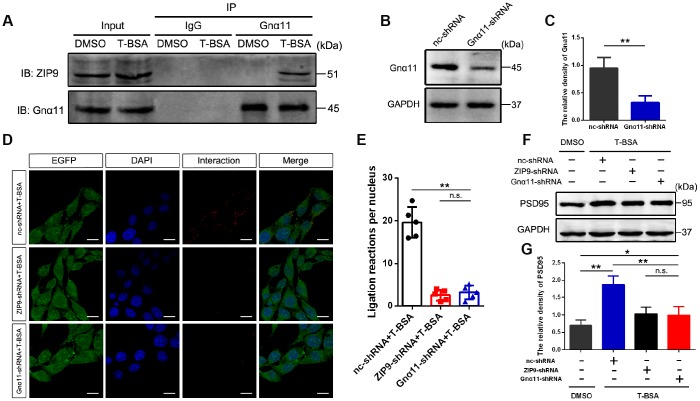
**ZIP9/Gnα11 interactions mediate the effect of T-BSA on PSD95 expression.** (**A**) Co-IP for the interaction between ZIP9 and Gnα11 induced by T-BSA. (**B** and **C**) Western blotting for the knockdown efficiency of Gnα11 protein in HT22 cells transfected with nc-shRNA or Gnα11-shRNA. (**D** and **E**) Duolink^®^ proximity ligation assay for the interaction between ZIP9 and Gnα11 induced by T-BSA pre-treated with nc-shRNA, ZIP9-shRNA or Gnα11-shRNA. (**F** and **G**) Western blotting for PSD95 expression induced by T-BSA in HT22 cells pre-treated with nc-shRNA, ZIP9-shRNA or Gnα11-shRNA. (n = 5; n.s.: no-significant; * *P* < 0.05; ** *P* < 0.01).

We used Co-IP to determine the interaction of ZIP9 and Gnα11 detected in HT22 cells following T-BSA treatment (Figure 3A). Western blotting analysis confirmed that Gnα11-shRNA significantly reduced Gnα11 protein level compared with the cells that received the nc-shRNA treatment (Figure 3B and 3C). We then used PLAs which detecting protein-protein interactions in fixed cells [26] to further confirm the direct interaction between ZIP9 and Gnα11. The red color in Figure 3D represented with a strong ZIP9/Gnα11 interaction signals observed in HT22 cells treated with nc-shRNA plus T-BSA. However, knockdown of ZIP9 or Gnα11 expression with ZIP9-shRNA or Gnα11-shRNA significantly reduced this interaction compared with nc-shRNA treatment (Figure 3D and 3E). Meanwhile, treatment with either ZIP9-shRNA or Gnα11-shRNA significantly abolished T-BSA-induced upregulation of PSD95 protein expression levels compared with the nc-shRNA plus T-BSA group (Figure 3F and 3G).

### T-BSA upregulated the phosphorylation levels of Erk1/2 and eIF4E by ZIP9/Gnα11

**Figure 4:**
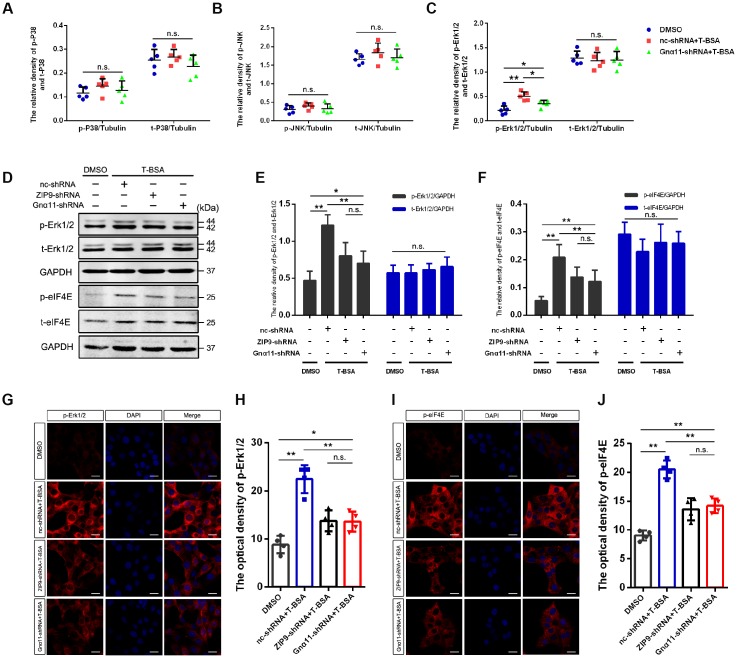
**T-BSA upregulated the phosphorylation level of Erk1/2 and eIF4E through ZIP9/Gnα11.** (**A**–**C**) The ratio of phosphorylated/total levels of P38 (**A**), JNK (**B**), and Erk1/2 (**C**) detected in HT22 cells pre-treated with nc-shRNA or Gnα11-shRNA using Flowmetric Luminex xMAP^®^ assay (n=5). (**D**–**F**) Western blotting for the phosphorylated/total levels of Erk1/2 and eIF4E induced by T-BSA pre-treated with nc-shRNA, ZIP9-shRNA, or Gnα11-shRNA (n=5). (**G**–**J**) Immunofluorescence staining for the phosphorylation level of Erk1/2 and eIF4E induced by T-BSA pre-treated with nc-shRNA, ZIP9-shRNA or Gnα11-shRNA (n=4, scale bars = 20 μm). (n.s.: non-significant; * *P* < 0.05; ** *P* < 0.01).

To determine the downstream molecular mechanisms following activation of ZIP9/Gnα11, Flowmetric Luminex xMAP^®^ assay was carried out to examine the effect of T-BSA on members in the mitogen-activated protein kinase (MAPK)-related signaling pathways in HT22 cells. T-BSA treatment did not change total Erk1/2 expression level but significantly increased phosphorylated Erk1/2 level compared with that in DMSO-treated HT22 cells. Furthermore, T-BSA treatment has little effect on either total levels or phosphorylated levels of P38 and JNK (Figure 4A–4C). Knockdown of Gnα11 expression with shRNA dramatically inhibited T-BSA-induced increase in phosphorylated-Erk1/2 (p-Erk1/2) level. The role of ZIP9/Gnα11 in T-BSA-induced increase in p-Erk1/2 was further confirmed by western blot and immunofluorescent staining analysis. The results showed that the p-Erk1/2 levels in ZIP9-shRNA plus T-BSA group and Gnα11-shRNA plus T-BSA group were significantly lower than those in nc-shRNA plus T-BSA group (Figure 4D–4H).

The finding that CHX treatment removed T-BSA-induced PSD95 expression suggest that this action may involve the regulation of the translation process. The eukaryotic translation initiation factor (eIF) 4E, which involved in the recruitment of the ribosome to the mRNA cap structure, integrates input from Erk1/2 signaling to regulate the translation of a subset of mRNAs [27, 28]. Western blotting and immunofluorescent staining analysis showed that knockdown of ZIP9 and Gnα11 expression significantly reduced phosphorylated-eIF4E (p-eIF4E) level compared with the nc-shRNA plus T-BSA group (Figure 4D–4J). Quantification of eIF4E immunoreactivity revealed that T-BSA treatment upregulated the p-eIF4E by ZIP9/Gnα11 in HT22 cells.

### Erk1/2 activation is required for T-BSA-induced eIF4E phosphorylation and PSD95 expression

**Figure 5:**
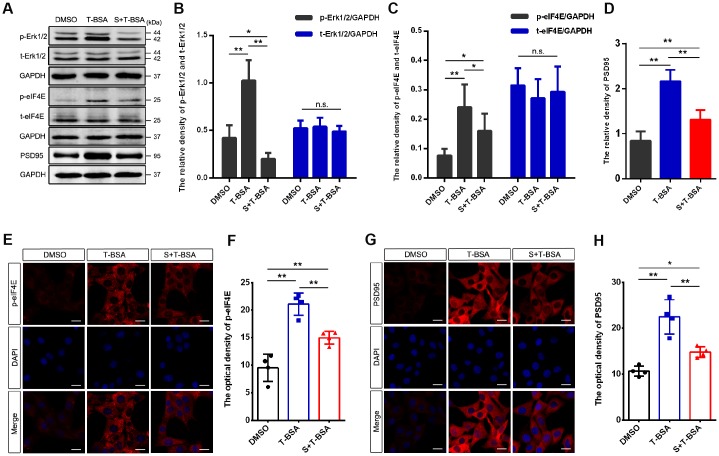
**Erk1/2 activation is required in T-BSA-induced eIF4E phosphorylation and PSD95 expression.** (**A**–**D**) Western blotting for the phosphorylated /total levels of Erk1/2 and eIF4E and the level of synaptic protein PSD95 induced by T-BSA in HT22 cells pre-treated with the Erk1/2 inhibitor SCH772984 (S, 100 nM) for 2 h (n=5). (**E**–**H**) Immunofluorescence staining for p-eIF4E and PSD95 induced by T-BSA pre-treated with S (n=4, scale bar = 20 μm). (n.s.: non-significant; * *P* < 0.05; ** *P* < 0.01).

To assess if Erk1/2 signaling is involved in T-BSA-induced PSD95 expression, we used the specific Erk1/2 inhibitor SCH772984. As expected, western blotting analysis showed that pretreatment with SCH772984 blocked Erk1/2 phosphorylation in T-BSA-treated HT22 cells (Figure 5A and 5B). We thus further determined the effect of SCH772984 on eIF4E phosphorylation and PSD95 expression induced by T-BSA. Western blotting and immunofluorescent staining analysis demonstrated that HT22 cells treated with SCH772984 plus T-BSA exhibited significantly lower eIF4E phosphorylation compared to that in cells treated with T-BSA alone (Figure 5A and 5C, 5E and 5F).

Western blotting and immunofluorescent staining analysis demonstrated that HT22 cells treated with SCH772984 plus T-BSA exhibited significantly lower PSD95 protein level compared with that in cells treated the T-BSA alone (Figure 5A and 5D, 5G and 5H). These results revealed that eIF4E phosphorylation and PSD95 expression induced by T-BSA depend on Erk1/2 activation.

## DISCUSSION

Androgen that is produced and secreted by Leydig cells in the testis plays critical roles in the development and maintenance of the male phenotype and reproductive function [29]. In addition, emerging evidence supports a pivotal role of androgen in cognitive function, especially its effect on synaptic plasticity that is involved in learning and memory and cognitive function [30]. The volumetric density of spine synapses (number of spine synapse/µm^3^) in the hippocampus CA1 area was found to be significantly reduced (40%) in gonadectomized male non-human primates compared to their intact counterparts [31]. On the other hand, application of testosterone, a major endogenous androgen, was found to rapidly increase the dendritic spine density in rat and mouse hippocampus [11, 32]. Our previous studies also revealed that testosterone rapidly increased the level of the synaptic protein PSD95 along with an increase in the dendritic spine density in the hippocampus of SAMP8 mice [13]. However, the underlying mechanism remains unclear. Synaptic protein PSD95, a member of the membrane-localized guanylate kinase (MAGUK) family, is a scaffold protein that assembles a complex of glutamate receptors, ion channels, and signaling proteins. Several studies have demonstrated that the levels of PSD95 in AD models and patients during the course of the disease were reduced, which was attributed to a relatively fast genetically driven pathological progression [33, 34]. In the present study, we demonstrated that T-BSA rapidly increased PSD95 expression in HT22 cells, which was confirmed to occur through a transcription-independent mechanism. Our finding is strongly supported by previous studies showing that the maintenance and regulation of synaptic proteins is regulated, in part, by local mRNA translation in neuronal dendrites [35, 36]. This regulator mechanisms enable neurons to exhibit rapid signaling by processing information in a precise and, sometimes, spatially restricted manner [35].

Since the genomic mechanism underlying androgen action cannot account for the rapid effects of T-BSA on PSD95 expression, our findings suggest a non-genomic pathway underlying T-BSA-induced PSD95 expression. Indeed, over the past two decades, evidence has accumulated to implicate a rapid role of androgen through non-genomic actions [32, 37]. Because T-BSA is a membrane impermeable macromolecule complex, we speculated that T-BSA-induced rapid induction of PSD95 expression was mediated by the membrane-binding sites. The notion is supported by the observation that T-BSA-FITC binding signals in HT22 cell membranes. The existence of androgen membrane-binding sites has been postulated by many researchers based on the detection of specific androgen binding to plasma membranes in different cell types including colon tumor cells [38], breast cancer cells [39], prostate cancer cells [40] and Sertoli cells [41]. However, the nature of these binding sites remains controversial.

Several studies have demonstrated that the AR mediates the rapid non-genomic actions of androgen. AR is a member of the nuclear receptor superfamily and acts as a ligand-dependent transcription factor, which is considered to be either 1) located in the cytoplasm and then translocated to the nucleus upon activation, 2) tethered to the inside of the plasma membrane, or 3) retained in the nucleus until free androgens enter and activate specific DNA response elements. However, Morrill et al. [20] found that the cytosol-nuclear receptors for androgen and estrogen (ER) contain two transmembrane helices within their ligand-binding domains using computational methods analyzing peptide receptor topology. AR as well as both the α and β subtypes of ER are found in the hippocampal neurons in both the nucleus [42, 43] and extra-nuclear sites including the plasma membrane, mitochondria and synaptic vesicles [43–46]. A recent study has shown that testosterone binds to AR located on cell membrane and results in a rapid activation of Src kinase, which is critical for spermatogenesis in Sertoli cells, an effect is eliminated by flutamide [37]. These findings provide direct evidence for the existence of membrane-localized AR. In this study pre-treatment HT22 cells with flutamide, had no effect on T-BSA-induced PSD95 expression, suggesting that membrane-localized AR did not mediate this process.

In addition to membrane-localized steroid receptors, GPCRs have also been shown to play an important role in rapid non-genomic actions of steroids, such as G protein-coupled estrogen receptor 1 (GPER1, also known as GPR30) for estrogen [47, 48] and membrane progestin receptor (mPR) for progesterone [49–51]. Notably, GPER1 has been identified as one of the main estrogen-sensitive receptors responsible for the rapid enhancement of hippocampal synaptic plasticity [47, 48]. Recently, Thomas and colleagues [52] reported the cloning and expression of a cDNA from Atlantic croaker ovaries encoding a seven-transmembrane protein that resulted in the rapid, cell surface-mediated androgenic inhibition of estradiol secretion. This protein showed 81–93% amino acid sequence identity with zinc transporter ZIP9 subfamily members, indicating that it is a ZIP9 protein. In parallel, Thomas et al. confirmed that ZIP9 has a similar function in human prostate and breast cancer cells [53]. Here, we further determined the role of ZIP9 in mediating the androgen’s non-genomic pathways on synaptic plasticity. Upon knockdown of ZIP9 expression using shRNA, the effect of T-BSA on PSD95 expression induction was inhibited. These results suggest that ZIP9 serves as an androgen membrane-binding site to mediate this process.

ZIP9 is a zinc transporter and is known to act as a mediator of signaling events through G protein-Gnα11. Despite these pivotal properties, its physiological and pathophysiological functions have not yet been thoroughly investigated. Using Co-IP and PLA, we confirmed that T-BSA caused a direct interaction between ZIP9 and Gnα11 in HT22 cells. However, when ZIP9 or Gnα11 expression was knocked down, the interaction was significantly inhibited, demonstrating that T-BSA induces this interaction, and the consequent complex activates intracellular signal transduction molecules. Gnα11 belongs to the Gq protein class of G proteins, which activates many signaling pathways, including the MAPK [54, 55]. The MAPK signaling pathway reacts to multiple intracellular and extracellular signals, such as hormones, cytokines, mitogens, growth factors and neurotransmitters. This pathway has also been linked to the regulation of synaptic plasticity and learning and memory [56, 57], and contributes to the pathology of diverse human diseases, including neurodegenerative disorders such as AD [58, 59]. Moreover, the MAPK pathway exerts significant roles in the non-genomic actions of steroids [60–62]. The MAPK pathway involves 3 distinct cascades, including the Erk1/2, JNK, and P38 MAPK pathways. In this study, we demonstrated that the phosphorylation of Erk1/2 is mainly responsible for the ZIP9/Gnα11-mediated androgen non-genomic pathway. Several recent reports have implicated Erk1/2 in regulating the phosphorylation of the translation initiating factor, eIF4E, to control the synthesis of synaptic proteins and synaptic plasticity [27]. We discovered that T-BSA upregulated eIF4E phosphorylation level by ZIP9/Gnα11 and that inhibition of Erk1/2 activity partially blocked this effect, indicating that additional pathways, such as mammalian target of rapamycin (mTOR) signaling, also lead to synaptic synthesis of PSD95 [36].

In conclusion, through utilizing a series of morphological and molecular biology techniques, we confirmed that T-BSA affects translation process via the ZIP9-mediated Gnα11/Erk1/2/eIF4E pathway, then rapidly increases the level of synaptic protein PSD95 in HT22 cells. This study not only provides a theoretical basis for understanding of the androgen non-genomic pathway regulating synaptic plasticity, but also further highlights new potential therapeutic targets for development of medication to treat learning and memory disorders such as AD. However, the findings from this study are based on the data collected from one cell line. Thus, further studies are warranted to validate the findings in other cell lines and in animal models.

## MATERIALS AND METHODS

### Cell culture

Immortalized mouse hippocampal HT22 cells were maintained in Dulbecco’s modified Eagle medium (DMEM) with 10% fetal bovine serum (FBS) and a 0.2% penicillin/streptomycin solution in an H_2_O-saturated 5% CO_2_ atmosphere at 37°C. The cells were digested with 0.25% trypsin at 37°C from 10 cm culture dishes at 70–80% confluence. The cells were then seeded at 50,000 cells/cm^2^ and cultured for another day in 6-well plates for protein/mRNA assays or were mounted on coverslips in 12-well plates for immunofluorescence staining.

### Reagents

T-BSA (cat#: T3392) and fluorescein isothiocyanate-conjugated T-BSA(T-BSA-FITC) (cat#: T5771) were purchased from Sigma-Aldrich (MO, USA). BSA-FITC (cat#: SF063) was purchased from Solarbio (China). Actinomycin D (Act D; cat#: HY-17559) and Cycloheximide (CHX; cat#: HY-12320) were purchased from MedChemExpress (NJ, USA). Flutamide (F; cat#: F0663) and Dimethyl sulfoxide (DMSO; cat#: D0798) were purchased from Tokyo Chemical Industry Co., Ltd (Japan). SCH772984 (cat#: S7101) was purchased from Selleck Chemicals (TX, USA). Rabbit anti-PSD95 (cat#: ab18258), rabbit anti-phospho-eIF4E (S209) (cat#: 76256), rabbit anti-eIF4E (total) (cat#: 33766), rabbit anti-GAPDH (cat#: ab9485) antibodies were purchased from Abcam (MA, USA). Rabbit anti-phospho-Erk1/2 (Thr202/Tyr204) (cat#: 9101) and mouse anti-Erk1/2 (total) (cat#: 9107) antibodies were purchased from cell signaling technology (MA, USA). Rabbit anti-ZIP9 antibody (cat#: GTX31817) was purchased from GeneTex (CA, USA). Mouse anti-Gnα11 (cat#: sc-390382) and normal mouse IgG (cat#: sc-2025) antibodies were purchased from Santa Cruz Biotechnology (MA, USA).

### Western blotting

Protein samples were isolated in 200 μl of RIPA buffer supplemented with protease and phosphatase inhibitors (Complete Protease Inhibitor Cocktail, cat#:04693116001 and PhosSTOP, cat#:04906845001, Roche, Switzerland). The homogenates were cleared by centrifugation at 12,000 *g* for 20 min at 4 °C. Total protein concentration was evaluated using BCA Protein Assay reagent kit (cat#: PC0020, Solarbio, China) and an Infinite F200 (TECAN, Switzerland) plate reader. Protein samples were mixed with 5X Laemmli buffer. After boiling, 25 μg of protein was loaded onto a 10% sodium dodecyl sulphate-polyacrylamide gel for protein separation and electro-transferred onto polyvinylidene fluoride membranes. The membranes were then blocked with 5% milk in TBS-T (0.1% Tween-20) and incubated with the following diluted primary antibodies overnight at 4 °C (rabbit anti-PSD95, 1:1,000; rabbit anti-phospho-Erk1/2 (Thr202/Tyr204), 1:2,000; rabbit anti-ZIP9, 1:1,000; rabbit anti-phospho-eIF4E (S209), 1:1,000; rabbit anti-eIF4E (total), 1:1,000; rabbit anti-GAPDH, 1:2,000; mouse anti-Erk1/2 (total), 1:1,000; mouse anti-Gnα11, 1:1,000). The membranes were then washed five times in TBST for 5 min each, and blotted with the secondary antibody (rabbit IgG (H&L) Antibody Dylight™ 800 Conjugated, cat#: 610-445-002 and mouse IgG (H&L) Antibody Dylight™ 680 Conjugated, cat#: 610-144-002, 1:10,000, ROCKLAND, USA) for 2 h at room temperature. After washing again, reactions were visualized and analyzed using an Odyssey IR fluorescence scanning imaging system (LI-COR, USA).

### Cell-surface labelling with T-BSA-FITC

To detect the presence of membrane androgen-binding sites, HT22 cells were incubated with10^−5^ M T-BSA-FITC for 20 min at 37 °C. The medium was then aspirated, and the cells were fixed with 1 ml ice-cold 4% paraformaldehyde (PFA) for 10 min. After washing five times for 5 min each in 0.01 M phosphate-buffered saline (PBS), the coverslips were mounted with Vectashield-DAPI, and then gently placed over the slide. BSA-FITC was used as the control for detecting non-specific FITC signals. Images were obtained by an inverse Olympus FV1200 microscope equipped with the corresponding fluorescence system (Olympus, Hamburg, Germany) and were analyzed using Image Pro Plus 6.0 to quantify the fluorescence intensity.

### Immunofluorescence staining

The cells were fixed with 1 ml ice-cold 4% PFA for 10 min at room temperature. After washing with 0.01 M PBS, the cultured cells on coverslips were blocked with PBS-T (0.5% Triton X-100) containing 5% donkey serum for 60 min at room temperature, followed by incubation with primary antibodies diluted in 0.01 M PBS overnight at 4 °C (rabbit anti-PSD95, 1:200; rabbit anti-phospho-Erk1/2 (Thr202/Tyr204), 1:200; rabbit anti-phospho-eIF4E (S209), 1:200). After washing, the coverslips were incubated with secondary antibodies (Donkey anti-Rabbit IgG (H+L) Highly Cross-Adsorbed Secondary antibody Alexa Fluor® 594, 1:1,000, cat#: A-21207, Thermo Scientific™) for 2 h at room temperature and mounted with Vectashield-DAPI after a final wash. Images were acquired with an inverse Olympus FV1200 microscope equipped with the corresponding fluorescence system and were analyzed using Image Pro Plus 6.0 to determine the optical density for quantitative analysis.

### RNA isolation and reverse transcription-polymerase chain reaction (RT-PCR)

Total RNA from the cells was extracted using the SV Total RNA Isolation System (cat#: Z3100, Promega, Germany), which was used to synthesize cDNA with the GoScript™ Reverse Transcription System (cat#: A5001, Promega, Germany). Total RNA (2 μg) was used in reverse transcription reactions at a total volume of 25 μl with the following three-step incubation: 25 °C for 5 min, 42 °C for 60 min, and 75 °C for 15 min. Quantification of mRNA levels was performed using quantitative PCR with 2×Taq PCR MasterMix (cat#: KT201, TIANGEN, China). Samples were incubated at 94 °C for 3 min, followed by 40 cycles of denaturation at 94 °C for 30 s, annealing at 57 °C for 30 s, and cDNA extension at 72 °C for 45 s. After the amplification cycles, a final extension step at 72 °C was performed for 10 min. The results for the target gene were normalized to the level of *GAPDH* for quantifying relative mRNA expression levels. The genes were amplified using the following primers: *PSD95* forward, 5′TACCAAAGACCGTGCCAACG3′, and reverse 5′CGGCATTGGCTGAGACATCA3′; *GAPDH* forward, 5′CCGGTGCTGAGTATGTCGTG 3′, and reverse 5′TGGTCATGAGCCCTTCCACA3′.

### Knockdown of ZIP9 and Gnα11 with RNA interference

Short hairpin RNA (shRNA) targeting *ZIP9* (5′-ATTGTGTTCGTGGCAATAA-3′) and *Gnα11* (5′-ACTCACACTTGGTCGATTA-3′) were packaged into lentiviruses (Genechem Inc.) for transfection in the cells at 30% confluence. The cells were maintained in 0.5 ml DMEM containing 2% FBS with ZIP9-shRNA, Gnα11-shRNA, or the negative control LVCON077 (hU6-MCS-Ubiquitin-EGFP-IRES-puromycin) for 12 h, respectively. After replacing the medium with 1 ml DMEM containing 2% FBS, the cells were cultured for another 72 h. The knockdown efficiency was assessed by western blotting as described above.

### Co-immunoprecipitation (Co-IP)

After removing the culture medium, cells were washed once with ice-cold 0.01 M PBS and IP Lysis/Wash Buffer was added, followed by incubation on ice for 5 min. The homogenates were cleared by centrifugation at 12,000 *g* for 20 min at 4 °C. The total protein concentration was evaluated using BCA Protein Assay reagent kit and an Infinite F200 plate reader. The mouse anti-Gnα11 antibody or a control mouse IgG was used for immunoprecipitation (IP) and incubated overnight at 4°C to form the immune complex. The Pierce Protein A/G Magnetic Beads of Pierce™ Classic Magnetic IP/Co-IP Kit (cat#: 88804, Thermo Scientific™) were washed twice with ice-cold IP Lysis/Wash Buffer. The antigen sample/antibody mixture was then added to the tube containing the pre-washed magnetic beads and incubated at room temperature for 1 h with mixing. After washing the beads three times, the eluted proteins were subjected to SDS-PAGE and western blotting with rabbit anti-ZIP9 antibody (1:1,000) and mouse anti-Gnα11 antibody (1:1,000).

### Proximity ligation assay (PLA®)

The cells were fixed with 1 ml ice-cold 4% PFA for 10 min. After washing, the cultured cells on coverslips were permeabilized with PBST and then one drop of Duolink II blocking solution of the Duolink^®^ PLA^®^ Kit (Sigma-Aldrich, cat#: DUO92008, Germany) was added to each well and incubated for a further 1 h at 37 °C. Subsequently, 20 μl of rabbit anti-ZIP9 antibody (1:200) and mouse anti-Gnα11 antibody (1:200) were added to each coverslip, and the cells were incubated overnight at 4 °C and washed. A total of 40 μl minus and plus PLA probes of the kit were then added to each coverslip and incubation proceeded for 1 h at 37 °C. After washing, 40 μl of ligation-ligase solution was added to each coverslip and incubation continued for 30 min at 37 °C, followed by the addition of 40 μl amplification-polymerase solution and incubation for 100 min at 37 °C in the dark. The coverslips were mounted with Vectashield-DAPI, and then gently placed over the slide. Images were obtained by an inverse Olympus FV1200 microscope and analysed using Image J to determine the number of ligation reactions per DAPI-stained cell.

### Flowmetric Luminex xMAP® analysis

Protein samples were isolated in 200 μl of RIPA buffer supplemented with protease and phosphatase inhibitors. The homogenates were cleared by centrifugation at 12,000 *g* for 20 min at 4 °C, and the total protein concentration was evaluated using BCA Protein Assay reagent kit and an Infinite F200 plate reader. Aliquots of the supernatant were collected and stored at −80 °C until analysis using. Intracellular Bead-Based Multiplex Assays (cat#: 48-619MAG, 2-Plex Phospho/Total Erk Magnetic bead kit; cat#: 48-624MAG, 2-Plex Phospho/Total p38 Magnetic Bead Kit; cat#: 48-622MAG, 2-Plex Phospho/Total JNK Magnetic Bead Kit; cat#: 46-713MAG, Total β-Tubulin Magnetic Bead MAPmate™, Merck Millipore, USA) with Luminex technology. This method enables the simultaneous relative quantitation of multiple phosphorylated or total proteins in cell lysate samples using the Milliplex Multi-Analyte Profiling system.

### Statistical analysis

All data were analysed with SPSS 21.0 statistical software (SPSS Inc., Chicago, IL, USA). Direct comparisons between two groups were performed using the two-tailed Student *t* test. Multiple group comparisons were made with one-way analysis of variance with post-hoc Fisher’s *LSD* test. The results are expressed as the mean ± SEM. Statistical significance was defined as *P < 0.05 or **P < 0.01, respectively.
